# Azo-Dye-Functionalized Polycarbonate Membranes for Textile Dye and Nitrate Ion Removal

**DOI:** 10.3390/mi13040577

**Published:** 2022-04-07

**Authors:** Carrie Cockerham, Ashton Caruthers, Jeremy McCloud, Laura M. Fortner, Sungmin Youn, Sean P. McBride

**Affiliations:** 1 Department of Civil Engineering, Marshall University, Huntington, WV 25755, USA; cockerham2@marshall.edu (C.C.); youns@marshall.edu (S.Y.); 2 John T. Hoggard High School, 4305 Shipyard Boulevard, Wilmington, NC 28403, USA; ashton.caruthers@nhcs.net; 3 Department of Curriculum Instruction & Foundations, Marshall University, Huntington, WV 25755, USA; 4 Department of Mechanical Engineering, Marshall University, Huntington, WV 25755, USA; mccloud54@marshall.edu (J.M.); fortner40@marshall.edu (L.M.F.); 5 Department of Physics, Marshall University, Huntington, WV 25755, USA; 6 Spring Valley High School, 1 Timberwolf Drive, Huntington, WV 25704, USA

**Keywords:** dye separation, membrane filtration, rejection, flow rate, decolorization, pollution, textile wastewater, fabric treatments, hysteresis, azo dye

## Abstract

Challenges exist in the wastewater treatment of dyes produced by the world’s growing textiles industry. Common problems facing traditional wastewater treatments include low retention values and breaking the chemical bonds of some dye molecules, which in some cases can release byproducts that can be more harmful than the original dye. This research illustrates that track-etched polycarbonate filtration membranes with 100-nanometer diameter holes can be functionalized with azo dye direct red 80 at 1000 µM, creating a filter that can then be used to remove the entire negatively charged azo dye molecule for a 50 µM solution of the same dye, with a rejection value of 96.4 ± 1.4%, at a stable flow rate of 114 ± 5 µL/min post-functionalization. Post-functionalization, Na^+^ and NO3^−^ ions had on average 17.9%, 26.0%, and 31.1% rejection for 750, 500, and 250 µM sodium nitrate solutions, respectively, at an average flow rate of 177 ± 5 µL/min. Post-functionalization, similar 50 µM azo dyes had increases in rejection from 26.3% to 53.2%. Rejection measurements were made using ultraviolet visible-light spectroscopy for dyes, and concentration meters using ion selective electrodes for Na^+^ and NO3^−^ ions.

## 1. Introduction

Colors have been a part of everyday life since the dawn of human existence. Prior to the mass synthetic coloring era in the textile industry, beginning in the middle of the 19th century, for tens of thousands of years, humans have been using some form of natural surface modification to alter the color of objects for a wide variety of purposes [1,2,3,4,5]. With the age of vibrant synthetic colors in the middle of the 19th century came the understanding that the success of the industrial dying process is dependent upon the type of dye being used and the type of substrate to be colored. Given the desired combinations, several different mechanisms, including physical adsorption, mechanical retention, ionic processes, van der Waals forces, hydrogen interactions, and covalent bonding, could be responsible for successfully attaching dye molecules to a surface [6,7]. Currently, textile dyes have found applications in a wide variety of industries, including medicine, ink, cosmetics, temporary hair dye, food, pharmaceuticals, and paper [8,9,10,11,12,13,14,15,16]. Creating a vibrant, colorful world, through the use of synthetic dyes, has come at a devastating cost. In 1978, nearly half a century ago, it was estimated that the worldwide production of organic dyes per year was at 450,000 tons, with 9000 tons of dye lost in the form of effluents in our waterways during the manufacturing process, and approximately 41,000 tons were discharged in effluents in the application processes of these dyes [17,18,19,20]. Currently, it is estimated that the annual production of synthetic dyes alone is over 7 × 10^5^ metric tons, with an estimated 2.8 × 10^5^ tons of textile dyes being discharged from the textile industry each year [21,22]. Textile effluents are complicated matrices of many different components. In the estimated 200 million liters of effluents produced worldwide each year by the textile industry, 72 toxic chemicals have been identified [23,24,25].

The health effects on the global ecosystem and specifically on humans due to the discharge of textile wastewater that is either unsuccessfully treated, partially treated, or untreated are vast [16,17,22,26,27,28,29,30,31,32,33,34,35,36]. Once such cytotoxic, genotoxic, carcinogenic, and mutagenic dyes have entered the human body, they can cause DNA damage and cancer [37]. With these health and ecological consequences coming into focus in more recent years, some countries have banned the use of certain azo dyes and have established environmental legislation that prompts textile industries to, at a minimum, eliminate color from their dye containing effluents before the discharge into waterways (concentrations of 1 mg/L or less are highly visible for some dyes); however, some countries, such as India, Pakistan, and Malaysia, have had no mandatory emission limits, only recommended limits [6,27,38,39]. To remove the toxic elements in the textile effluents in our waterways, and meet demands in legislation, the scientific community has observed a surge in research and the development of improved or new remediation and removal methods ranging from electrochemical, adsorption, and desorption processes to advanced biodegradation and electron beam irradiation of azo dyes in wastewaters [22,25,27,40,41,42,43,44,45,46,47,48,49]. In recent years, significant strides have also been made in both experiment and simulation using polysulfone/polyetherimide ultrafiltration composite membranes for azo dye removal [50,51,52,53]. Overall, it remains a challenge to isolate a single efficient economical process that is effective at removing both the color and the toxic properties of textile wastewater that has been released into the environment; the more successful approach to purifying textile wastewater is a combination of treatment process, integrating several methods [6,27,41,47,54,55,56,57].

In this work, the substantive anionic direct azo dyes direct red 80, direct blue 14, and direct yellow 26 are used. Synthetic azo dyes are organic compounds that possess the chemical structure R−N=N−R, and make up approximately 50–70% of all available commercial dyes produced and used in industry [58,59,60]. The R groups on either side of the double nitrogen bond (−N=N−), also known as the azo bond or chromophore of the molecule, are typically phenyl and/or naphthyl ring structures, which have some combination of amino (-NH_2_), chloro (-Cl), hydroxyl (-OH), methyl (-CH_3_), nitro (-NO_2_), or sulfonic acid sodium salt (-SO_3_Na) functional groups attached [61]. One of the main uses of azo dyes is to alter the color of various substrates such as those found in the modern, fabric-focused textile industry [62]. Depending upon the desired dye and textile combination used, it is estimated that 2–50% of the dye molecules used during the dying process are lost in the effluent and remain in textile wastewater, even after extensive treatment [63]. Recent estimates predict that treatment of approximately 20,000 kg of textiles per day leads to approximately 3106 L of wastewater [24,64]. Overall, each year, it is estimated that the textile industry produces about 80 billion clothing items, using up to 200 tons of water per metric ton of textiles produced, resulting in roughly 17–20% of the industrial water pollution crisis being due to fabric dyes and treatments [23,26,65]. Unfortunately, some effluent focused physical and chemical wastewater treatment processes are unable to completely remove azo dyes due to their color fastness, stability, and resistance to degradation; all these characteristics make them highly desired for the textile industry, but hard to remove from wastewater streams [27,40]. Traditional municipal or industrial wastewater treatment facilities, relying on the activated sludge processes alone, have similar problems of being ineffective at completely removing dyes during treatment; instead of removing them, they simply return to the environment mostly unaltered, while potentially lowering the activity of sludge in the biological treatment process and reducing further processing capabilities [61,66,67]. Even if this activated sludge process is followed by a chlorination treatment process or ultraviolet light (UV) treatment, these treatments only reduce the mutagenic effects of some azo dyes, they do not eliminate them [27,68]. In fact, if further treatment such as ozonation is not provided, the chlorination and UV processes by themselves should be avoided in some traditional municipal or industrial wastewater treatment facilities for effluents containing azo dyes, because such processes can produce compounds that can be more harmful to the environment than the dye itself [27,69,70]. The chemical structure of azo dyes makes them less susceptible to complete degradation via photolysis, microbial attack, aerobic biodegradation, and oxidative catabolism, but allows for metabolization under certain anaerobic conditions [27]. Even though decolorization via anaerobic azo dye reduction is possible, the process leaves behind the carcinogenic aromatic amines, which are slow to degrade further under the same anaerobic conditions [27,71,72,73].

The main purpose of this work is to demonstrate that porous track-etched polycarbonate membranes, functionalized with azo textile dyes themselves, can provide a high-efficiency pathway for successful removal and decolorization of textile dyes found in the wastewater streams of the textile industry. A secondary aim of this work illustrates that such azo-dye-functionalized filters can also help to reduce other charged contaminants, for example, sodium and nitrate ions found in other industrial waste streams, including agriculture. These two specific waste streams are significant. The dyeing and finishing portion of the textile industry is the second largest polluter of clean water in the world following the lead polluter, the agricultural industry [23]. The raw wastewater streams from both of these industries are complicated matrices with a variety of pollutants. As discussed earlier, one purification method cannot be used to solve all the problems associated with economically turning polluted, unusable wastewater back into freshwater. Therefore, future industrial versions of such azo-dye-functionalized filters, similar to those bench-scale ones investigated in this study, are envisioned to be eventually used in two locations. The first location is integrated in a combinatorial manner as finishing filters in existing wastewater treatment facilities for the removal of textile dyes, nitrate ions, and other charged contaminants of similar size. Ideally, they would be placed after traditional treatment processes, such as vertical bar screening, grit chambers, primary and secondary clarifiers, coagulation–flocculation, and activated sludge processes, etc., but prior to a final disinfection process (chlorination, ultraviolet light, or ozonation). The second location would be integrated in a combinatorial manner into textile dying water supply chains in factories, allowing for the original clean intake water to be reused before eventual discharge to a treatment facility, which in turn will potentially allow for recollection and reconcentration of unused dye to be recycled and used again for future dyeing. During the experimental runtime and concentrations over which the azo-functionalized filters were tested, they have been shown to be largely resistant to decreases in flow rates and additional fouling post-azo-dye-functionalization. Importantly, this new technique of decolorization of azo dyes using azo-dye-functionalized filters leaves the azo bond intact and filters out the entire dye molecule, resulting in no carcinogenic aromatic amine byproducts. In summary, these filters would allow the reuse of water, allowing for reconcentration of dyes for reuse, are less susceptible to fouling over time, and leave the azo bond intact post-filtering, overcoming some of the current challenges for membrane filtration of textile dyes [27,40,41,46,47,49].

With an ever-increasing global population estimated at 7.7 billion in 2019, with projections near ~11 billion people in the year 2100, comes an increased demand for economical sources of freshwater needed to sustain life [74]. Civilizations and industrialized regions across the world are increasing, but the amount of water on Earth will remain constant for geological time scales [75]. Only 0.26% of all water on Earth is easily accessible as freshwater in our lakes and rivers; thus, having access to freshwater is not only a natural, geographic problem, but is also a problem further compounded by the pollution of available freshwater sources in areas where freshwater scarcity is already an issue [76,77,78]. As a result, the scientific community has witnessed a revolution of new innovative emergent potential water purification technologies in the area of nanomaterials and nanostructures, including layers of porous self-assembled monolayers of gold nanoparticle membranes [79,80], layers of aligned carbon nanotubes with diameters less than 2 nm [81], and porous graphene sheets [82,83,84,85], with many other systems spanning this wide range [86,87,88,89,90,91,92,93]. All these recent novel advances in filtration membranes and separation technologies could someday be working towards the same common goal: purifying one of our most vital resources on Earth, water. As part of this work, the performance of azo-dye-functionalized filters will be compared with the recently developed ultrathin self-assembled nanoparticle (USANP)-functionalized filters, prepared on the exact same polycarbonate support substrate, to provide a comparison with recent state-of-the-art filtration technologies. With pore sizes reported to be ~1.7 nm [79], such USANP membranes are classified as nanofiltration (NF) membranes [94].

## 2. Materials and Methods

### 2.1. Materials

**Figure 1:**
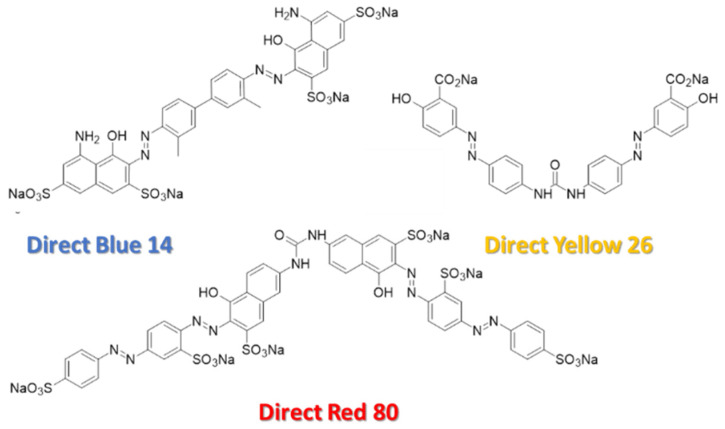
The chemical structure of the direct red 80, direct blue 14, and direct yellow 26, obtained from the ChemDraw database (PerkinElmer, Inc., Waltham, MA, USA).

**Figure 2:**
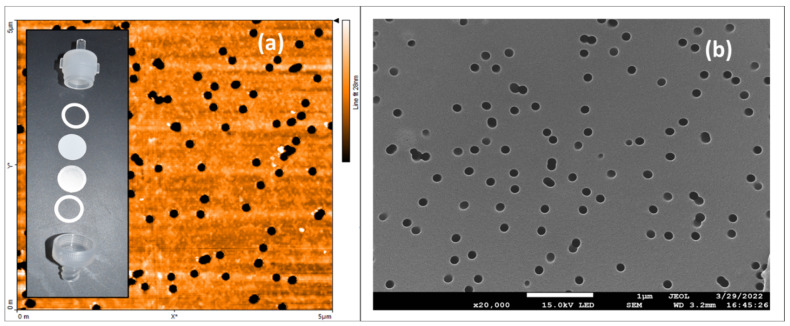
(**a**) Atomic force microscopy (AFM) image and (**b**) scanning electron microscopy (SEM) image of an unmodified polycarbonate filter. The color scale bar on the right of the AFM shows that the range in color from black to white spans only 28 nm. The polycarbonate filter in (**b**) was coated with a thin gold palladium coating for ease of SEM imaging. Inset: from top to bottom—the top end of a polypropylene housing with male Luer slip, silicon gasket, polycarbonate filter, photoetched stainless-steel screen, silicon gasket, and bottom end of polypropylene housing.

Several substantive anionic direct azo dyes were tested as functionalizing agents for commercially available polycarbonate filters; they were direct red 80 (Alfa Aesar, Ward Hill, MA, USA), direct blue 14 (Tokyo Chemical Industry Co., Ltd, Nihonbashi-honcho, Chuo-ku, Tokyo, Japan), and direct yellow 26 (Tokyo Chemical Industry Co., LTD, Nihonbashi-honcho, Chuo-ku, Tokyo, Japan); their chemical structures are shown in Figure 1. All dyes were used as received from the manufacturers. Sodium nitrate (98+%, Thermo Scientific, Waltham, MA, USA) was used as received from the manufacturer. The 25 µm-thick, 13 mm-diameter, hydrophilic track-etched polycarbonate filters, with 100 nm-diameter pores (Isopore Membrane Filters, MilliporeSigma, St. Louis, MO, USA) were used as received from the manufacturer prior to any functionalization processes. Figure 2 shows (a) an atomic force microscopy image (CoreAFM, Nanosurf, Liestal, Switzerland) and (b) a scanning electron microscopy image (JEOL 7200F-LV Field Emission SEM, Akishima, Tokyo, Japan) of a 5 µm × 5 µm area of an unmodified polycarbonate filter with 100 nm-diameter pores. The inset in Figure 2a shows the disassembled view of the standard polypropylene 13 mm-diameter filter housings and associated components designed to hold the polycarbonate filter.

**Figure 3:**
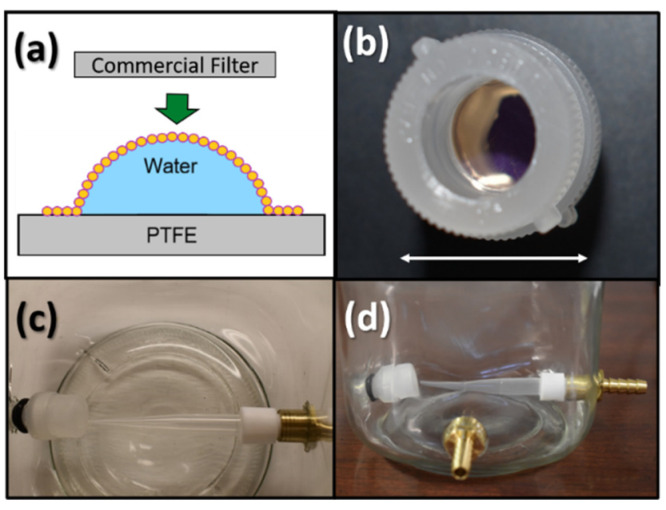
(**a**) The stamping process which transfers the USANP monolayers to the polycarbonate filters; (**b**) the machined polypropylene filter housings with USANP-functionalized polycarbonate filter installed, where the white arrow below indicates the length scale of 18 mm; (**c**) the top view looking down inside the custom filtration chamber; (**d**) the side view of the custom filtration chamber.

Some experiments in this study involve filters prefunctionalized with ultrathin self-assembled nanoparticle (USANP) monolayers prior to azo-dye-functionalization. Figure 3a shows how the USANP-functionalized filters were created by ‘stamping’ the flat commercial polycarbonate filter onto a single curved monolayer membrane of self-assembled gold nanoparticles deposited on a water interface. A more detailed description of the gold nanoparticle synthesis and ‘stamping’ process will follow in Section 2.2 and Section 2.3. All the water used in this study, unless indicated, was provided by a type 1 water source, which had resistivity equal to 18.2 MOhm, with less than 5 ppb total organic carbon (Direct Q 3-UV, Millipore Sigma, St. Louis, MO, USA). During the stamping process, the water droplet rests in a standard curved Petri dish that has been coated with a hydrophobic, chemically resistant polytetrafluoroethylene (PTFE) resin-based surface called Bytac (Saint-Gobain Performance Plastics, La Défense, Courbevoie, France), which is aggressively cleaned with ACS-grade toluene (Fisher Chemical, Waltham, MA, USA) and Kimwipes (Kimberly Clark Corporation, Irving, TX, USA) prior to deposition of the water in the stamping process.

The polycarbonate filters are designed to fit standard polypropylene 13 mm-diameter filter housings (Swinnex Filter Holder, MilliporeSigma, St. Louis, MO, USA); however, the polypropylene housings were modified in-house using a lathe (Grizzly Industrial, Inc., G4000, Washington, WA, USA) to remove unneeded polypropylene material to allow for direct flow of test fluid over the filter surface, as shown in Figure 3b. It should be noted that the filter installed in Figure 3b is a polycarbonate filter, functionalized with USANP layers, hence its purple-gold mirror-like appearance; if an unmodified polycarbonate filter was used, then the filter would appear white in Figure 3b. The filter housings are manufactured with female Luer lock inlets and male Luer slip outlets. The male Luer slip outlet is the one that is machined away so that the maximum exposed area of the polycarbonate filter (an open circle of 10 mm in diameter) can be visibly seen, as shown in Figure 3b (the male Luer slip on the top end of the housing can be seen in the inset of Figure 2a before being machined away). The polycarbonate filters in the polypropylene filter housing require the use of a stainless-steel photoetched support screen (MilliporeSigma, St. Louis, MO, USA) underneath the polycarbonate filter. The polycarbonate filter and steel support screen are compressed together in the housing using silicone gaskets (MilliporeSigma, St. Louis, MO, USA), specifically designed for the 13 mm Swinnex filter holders. The unassembled polycarbonate filter, polypropylene filter housing, associated support screen, and silicon gaskets are shown in the inset of Figure 2a.

Figure 3c,d show the top and side views, respectively, of the custom, in-house fabricated ~1 L glass filtration chamber, which typically houses 500 mL of test fluid. A lathe and milling machine (Grizzly Industrial, Inc., G0619, Washington, WA, USA) were used for fabricating holes in the glass chamber, as well as for machining Teflon stock and standard 0.25-inch brass barb hose fittings, which were held in place using quick-set 5 min epoxy (Gorilla Glue, Inc, Sharonville, OH, USA). To mate the polypropylene filter housing to the glass chamber, and make exchanging filters convenient, the threaded female portion of a standard disposable Luer lock syringe was used. It was first separated from the syringe, heavily roughened, and grooved on the outer surface, then epoxied inside a fabricated hole of matching diameter in the glass filtration chamber. A set of two O-rings, size #47 and #83 (Danco, Inc., Irving, TX, USA), were needed to complete the watertight compression seal between the glass chamber wall and the polypropylene filter housing. The bottom piece of polypropylene filter housing connects via a male Luer slip to a collection syringe, which in turn is connected to a vacuum pump ( ChoicestEquip, Zhoukou, Henan, China) with an inline drain valve fitting (Powermate, Vx 072-0001RP, South Burlington, VT, USA) to immediately equalize line pressure when the vacuum is stopped. The vacuum pump provides a pressure differential of 77.9 kPa to pull fluids through the polycarbonate filter. The white Teflon, as shown in Figure 3d, was machined to mate by compression into the brass fluid inlet fitting with the purpose of holding a 1000 µL standard polypropylene pipette tip, also by compression. The purpose of the polypropylene pipette tip was to create a fast-moving stream of test fluid that would be streamed onto the front surface of the functionalized polycarbonate filter to remove any large-scale concentration gradients in the test fluid at the surface of the functionalized filter. A peristaltic pump (DIPump550, Kamoer Fluid Tech (Shanghai) Co., Ltd., Shanghai, China) is connected to the filtration chamber via tubing and brass barb fittings. The peristaltic pump draws fluid out of the filtration chamber at 107.5 mL/min and deposits that fresh feed solution at high speed directly onto the polycarbonate filter via the narrowing polypropylene pipette tip. The high success of this setup, disrupting any large-scale concentration gradients near the surface of the filter, is shown in videos in Supplementary Materials S1 and S2. The videos show direct blue 14 dye being pumped into a freshwater solution showing the violent mixing within the chamber at the surface of the filter (Video S1 shows the side view and Video S2 shows the top view looking down into the chamber).

**Figure 4:**
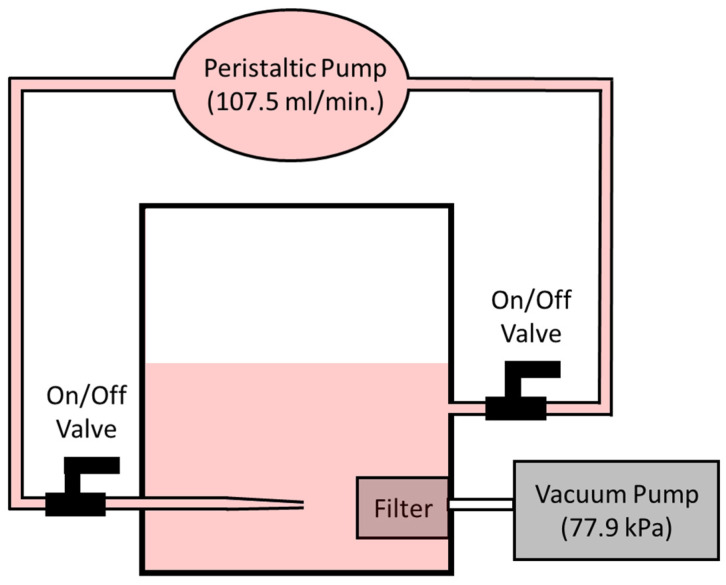
A diagram of the experimental filtration chamber setup.

A schematic of the setup is shown in Figure 4 for additional clarity. The red color represents the location of an azo dye or test fluid during functionalization or a filtration experiment. The ~500 mL of azo dye or test fluid completely surrounds the filter housing, which is located symmetrically within the filtration chamber. The filter housing is then connected to a vacuum pump to draw the fluid through the filter. A peristaltic pump creates a direct flow and supply of fresh feed directly at the filter surface through a narrowing high-velocity nozzle. Two simple on/off ball valves (Antylia Scientific, Masterflex Bioprocessing with Viton seals, Vernon Hills, IL, USA) provide an easy mechanism to exchange test fluids and clean the filtration chamber.

### 2.2. Gold Nanoparticle Synthesis

**Figure 5:**
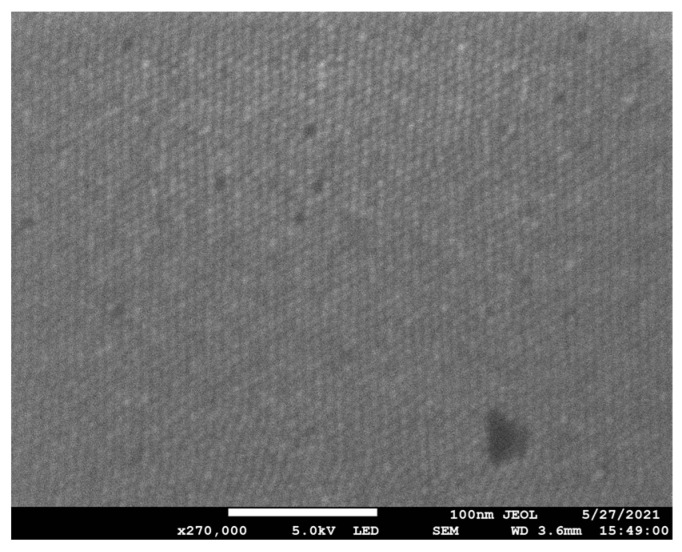
SEM image of a single layer of the gold nanoparticles that were synthesized and stamped onto a clean silicon surface. The white scale bar presented in the image represents 100 nm in length, providing gold nanoparticles of roughly 5 nm in diameter.

The general inverse micelle synthesis of several-nanometer-diameter gold nanoparticles, which in this work are used to functionalization polycarbonate filters with USANP monolayers, can be found in the literature [80,95,96,97]; however, a brief synopsis specific to this work will be provided for completeness. All glassware and components used in this synthesis were aggressively cleaned with a diluted 1% Alconox–tap water solution (Alconox, Inc., White Plains, NY, USA), rinsed with tap water, then sonicated for 20 min in a diluted 5% elma tec clean A4–tap water solution (Elma Schmidbauer GmbH, Singen, DEU). The glassware was then rinsed with tap water and then rinsed with copious amounts of type 1 water before being placed in an enclosed drying oven (100 L gravity convection oven, Thermo Scientific, Waltham, MA, USA). In a 25 mL standard bulb flask with a magnetic stirrer, a micelle solution was created using 20 mL of fresh sodium distilled toluene (started as ACS grade, Fisher Chemical, Waltham, MA, USA) added to 211.1 mg of didodecyldimethylammonium bromide (DDAB-99%) and this was used to dissolve 72.2 mg of gold chloride (AuCl_3_-99%) under room temperature sonication for 15 min (Branson 3800, Brookfield, CT, USA). Gold chloride was opened, handled, and resealed in an ultrahigh-purity nitrogen (Airgas, Radnor, PA, USA) environment in a custom in-house built dry box. A 72 µL aliquot, from a freshly made 9.35 M aqueous solution of sodium borohydride (NaBH_4_, Fisher Chemical, Waltham, MA, USA) that was left to age for 8.5 min, was quickly injected into the DDAB–AuCl_3_ solution, quickly sealed with a glass stopper, and violently shaken manually by hand. The process was carried out quickly and carefully so the byproduct synthesis gases were quickly released from the bulb flask every 3–5 s to prevent over pressurization of the 25 mL bulb flask, which can violently break apart due to the high reaction pressures. Violent manual shaking was carried out for approximately one minute, releasing the pressure periodically as described, before being placed on a magnetic stir plate (Thermo Scientific Cimarec stirring/hotplate, Waltham, MA, USA) for approximately 1 h and 15 min. Before leaving it to stir, is important that the 25 mL bulb flask not be closed completely. Next, the DDAB–AuCl_3_–NaBH_4_ solution was split equally into two 40 mL clean screw-top vials with Teflon septa by pipetting off the supernatant and leaving behind any unreacted solids in the bulb. A measure of 1 mL of dodecanethiol (DDT, 98%) was added to each 10 mL of solution in the 40 mL clean screw top vials and shaken violently by hand for several seconds. The vials were allowed to sit for 5 min before 20 mL of ethanol was added to each vial to precipitate the particles. The collection was aided by centrifugation (Eppendorf, 5804, Hamburg, Germany) at 1500 revolution per min (RPM) for 10 min. The supernatant was discarded, and the remaining particles were vacuum-dried (Buchi, R-100 System, Flawil, Switzerland) for 15 min (water bath at 35 °C and condenser coils at 10 °C, rotation speed 3.5/10) and then resuspended in 10 mL of sodium distilled toluene. With the addition of another 1 mL of DDT to each vial, the solution was heated under reflux at 170 °C for approximately 6 h. The vials were allowed to sit undisturbed after cooling down for one hour before the supernatant was then collected and placed into two separate clean screw top vials with Teflon septa. Unreacted solids were left in the bottom of the screw top vials. To remove synthesis byproducts, the supernatant was precipitated twice in ethanol (Pharmco-Aaper part of Greenfield Global, ACS grade, Toronto, ON, Canada), and centrifugation assisted in this process using 3500 RPM for 20 min for each cycle. After the second wash, the precipitate was dried under vacuum and redispersed in 10 mL of sodium-distilled toluene, which contained a 10^−4^ volume fraction of DDT. Each vial was then rotated in the water bath of the rotary evaporator at 35 °C for 5 min followed by a brief, several-second sonication to remove any particles from the side walls of the glass vials. Each vial was then centrifuged for 20 min at 3500 RPM and the supernatant was collected and used as the final product for USANP-functionalization. DDAB, AuCl_3_, and DDT were all from the same company (Acros Organics, Geel, Belgium). All chemicals, except the further sodium distillation of the ACS grade toluene, were used as received from the manufacturer. Sodium metal stored in mineral oil (Sigma Aldrich, St. Louis, MO, USA) was first cleaned by boiling in mineral oil and allowed to cool and solidify with any oxidation layer removed. The sodium was then rinsed excessively with toluene prior to cutting and dispersing in the toluene still (ChemGlass, Vineland, NJ, USA). Figure 5 shows a scanning electron microscope (JEOL 7200F-LV Field Emission SEM, Akishima, Tokyo, Japan) image of a single monolayer of the USANPs on an organic-solvent-cleaned and CO_2_-snow-jet-cleaned (Applied Surface Technologies, New Providence, NJ, USA) silicon surface (Electron Microscopy Sciences, Hatfield, PA, USA). These are the nanoparticles used in this study for USANP-functionalization of the polycarbonate filters. The particles are separated at a maximum by the 1.77 nm-long interdigitated dodecanethiol ligands bound to the nanoparticle surfaces [98].

### 2.3. Stamping

To make the self-assembled monolayers with the gold nanoparticles described in the previous section, 800 µL of type 1 water was placed on cleaned, chemically resistant Bytac in the center of a curved Petri dish. Then, 20 µL of the toluene that contained a 10^−4^ volume fraction of dodecanethiol, and the gold nanoparticles described in the previous section, were deposited at the three-phase contact line of the water droplet, initiating the self-assembly process [99,100]. If the Bytac surface was appropriately cleaned and the gold nanoparticles synthesis was successful, then once the nanoparticles solution was added, one should have expected to see quick, vigorous self-assembly at the air–water interface, at the apex of the water drop. If this vigorous self-assembly was not seen, and self-assembly occurred rather slowly with apparent pockets in the monolayer, and did not form a uniform, complete, continuous layer, especially at the apex of the drop, chances are that the Bytac surface or water was contaminated, the fresh gold nanoparticle solution was not washed well of DDT, there was too much DDT in solution, or the lifetime of the ligand-coated nanoparticle solution was expired. An expired solution, one that has simply aged, has the same assembly characteristics as a solution that has excessive DDT in the system. After 20 min, all toluene solution was evaporated, and then any partially hydrophilic surface, such as a clean piece of silicon or polycarbonate filter, was pushed into, or gently dropped on top of this single 5–8 nm-thick monolayer of nanoparticles. Highly smooth hydrophobic surfaces are less likely to attach by stamping. Successive layers can be applied by repeating this process. Between each stamping, the filters were placed in a vacuum for 5 min to remove any small quantities of residual water before the next layer was added. The filters, once stamped with the desired number of USANP layers, were first left to dry overnight, before being placed in a modified commercial polypropylene filter housing for testing.

For stamping, it was quickly realized that, due to the randomness of the cracks generated when taking the hemispherical area of the USANP monolayer and forcing it onto the flat surface of the polycarbonate commercial filters, similar flow rates with the exact same number of layers was not consistently achievable. Rather than keeping the number of layers on the filters constant, USANP-functionalized filters with similar starting water flow rates were employed. USANP-functionalized filters with 6–8 layers to yield 15 min pre-azo-dye-functionalization water flow rates in the range of 125–215 µL/min, with a standard deviation of less than 4% from the average nominal values were used.

### 2.4. Anionic Direct Azo-Dye-Functionalization Process

The azo-dye-functionalization process of the polycarbonate filters is a combination of simplicity, elegance, and irony. Azo dyes are a large contributor to the sources of freshwater pollution and, ultimately, are an important element to help remove them, as this work shows. To functionalize the polycarbonate filter, first, azo dye solutions are made at the desired concentrations. The structure of direct red 80, as shown in Figure 1, indicates that, when it dissociates in an aqueous solution, it separates into 6 positively charged sodium ions in solution, leaving the remaining dye molecule with 6 negatively charged sulfonate functional groups (SO^3−^) attached to the dye molecule, yielding its anionic character. Similar characteristics are seen for the direct blue 14 and direct yellow 26. When they dissociate in an aqueous solution, the dye molecules are left with 4 negatively charged sulfonate functional groups (SO^3−^) and 2 negatively charged carboxylate functional groups (COO^−^), respectively. A minimum of 15 min of water runs are collected pre- and post-azo-dye-functionalization to help with the characterization of the filters. Next, the desired direct azo dye is simply pulled through the porous polycarbonate filter under the supplied vacuum pressure. This starts the functionalization process, which is essentially running dye at a high concentration through the filter. As the charged dye molecules with different functional groups start to be pulled through the initially unmodified porous polycarbonate filter, the flow rate decreases and the rejection of the dye itself increases. As the data in this investigation shows, this behavior is hysteretic when examining the rejection as a function of increasing and decreasing concentration, indicating that some permanent or semi-permanent functionalization of the polycarbonate membrane is taking place. Filters are flushed for 15 min with water between changing dye concentrations to remove any unbound dye molecules. This azo-dye-functionalization process was carried out on both unmodified polycarbonate filters and polycarbonate filters already prefunctionalized with USANP membranes prior to the azo-dye-functionalization process. All azo-dye-functionalization was carried out at the native pH of the azo dye 1000 µM solutions (6.3 pH for direct red 80, Horiba LAQUA twin pH meter using glass electrode method, Kyoto, Japan) and at room temperature of 25 °C, without the addition of any mordant additives to prepare the polycarbonate or USANP surfaces for increased dye adsorption and/or bonding. In the textile industry, such anionic direct azo dyes have shown to predominately interact with cellulose fibers in fabrics via van der Waals, dipolar, and hydrogen-bonding intermolecular forces, but their interactions with unmodified polycarbonate and USANP monolayers of gold nanoparticles are not well documented [7,42].

### 2.5. Rejection and Flow Rate Measurements

An ultraviolet visible spectrometer (ND-1000 Spectrophotometer, NanoDrop Technologies, Inc., Wilmington, DE, USA) was used to measure the absorbance of direct azo dye solutions that did not undergo filtration, called the feed solutions, and those that did undergo filtration called the permeate solutions. The rejection of the azo dye molecules, R, was determined using the following equation:

(1) $$R=\left(1-\frac{\mathrm{Abs}.\;\mathrm{of}\;\mathrm{Permeate}}{\mathrm{Abs}.\;\mathrm{of}\;\mathrm{Feed}}\right)\times 100\%$$

The absorption of each sample was typically measured three times to ensure confidence in the measurements and no air bubbles are present in the small volume column of fluid used (~2.5 µL) for the spectroscopic measurement. Prior to determining the unknowns, all azo dye solutions used were tested for their absorbance as a function of concentration and all were determined to be well-defined, linear relationships; thus, the absorbance can easily be tied to the concentration of the test fluids. The greatest deviation from a zero y-intercept for the dyes was 1.7 × 10^−3^ on the absorbance scale and the worst r-squared value was 0.998. Calibration data and a plot of absorbance as a function of dye concentration for all azo dyes used are shown in the Supplementary Materials Figure S1. Direct blue 14 was the most intense color per given concentration, followed by direct red 80 and then direct yellow 26. A path length of 0.2 mm was used for direct blue 14 and direct red 80 at wavelengths of 583 nm and 544 nm, respectively. A larger path length of 1.0 mm was used for direct yellow 26 at 388 nm.

Using Equation (1) and ion-specific concentration meters (Horiba LAQUAtwin nitrate ion and sodium ion meters, Kyoto, Japan) to replace absorbance measurements, the rejection of the individual species of positively charged sodium ions and negatively charged nitrate ions were determined. Similarly, before measuring unknowns, the sodium nitrate solution at known concentrations were tested to ensure linearity in the concentration of ions as a function of solution concentration. There was a y-intercept deviation from 0 of 3.7 ppm in the ion concentration axis for the nitrate probe, which remained constant as a function of the solution concentration tested. The sodium meter had a 1.0 ppm offset in ion concentration, which remained constant as a function of solution concentration tested. Both r-squared values rounded to 0.999. Before each measurement, it was found necessary to perform multiple 2-point calibrations at 150 ppm and 2000 ppm iteratively for each meter, using calibration fluids to ensure the same level of accuracy found in the calibration plots. Calibration data and a plot of ion concentration as a function of sodium nitrate solution concentration are supplied in the Supplementary Materials Figure S2.

Only small aliquots of test fluid were pulled through the filter as the permeate, thus the 500 mL of fluid was considered an infinite reservoir of constant fresh feed solution. In theory, the absorbance of this 500 mL reservoir must increase if the filter is successful at removing dye molecules. Knowing this, each time a new dye concentration was used for direct red 80 at 1000 µM, a fresh feed sample was taken directly from the filtration chamber for each experiment, and was analyzed as the feed solution for that run to make sure the absorbance of the feed solution was well represented. As Table S1 demonstrates, no systematic increases in feed solution concentration were detected within the accuracy limits of the UV-Vis spectrometer over all experiments conducted for direct red 80, the main functionalization fluid used in this experiment.

Volumetric flow rate measurements were taken using a standard stopwatch and precision balance (Ohaus PX224 Pioneer Analytical Balance with 0.0001 g resolution, Parsippany-Troy Hills, NJ, USA).

### 2.6. Water Flow Rate Characterization of the Unmodified Polycarbonate Filters

Prior to any functionalization of the unmodified polycarbonate filters, in any experiment, water flow rates through each filter were measured to achieve a baseline for each filter. First, 5 measurements every 3 min were collected and analyzed. Unmodified filters had a flow rate of 355–390 µL/min with deviations from the average for each filter ranging from 0.1% to a maximum of 0.9% over this 15 min time period. The flow rates presented in this research might seem alarmingly small; however, the reader should keep in mind that the filters used only have a 3% active area (open area vs. closed area—this is exemplified in Figure 2, as most of the area is polycarbonate, not open pores) and the total area of the porous polycarbonate exposed in these experiments was confined to the area inside a 1 cm-diameter circle for the benchtop filters investigated. The design of these filters can be scaled up with greater porosity and larger flow rates, as discussed in the Results and Discussion Section. Flow rates for each filter prior to testing of any kind are very stable and indicate the high quality of commercial filter production, filter cleanliness, and water purity used in the experiments. This water flush helped to ensure that the starting flow rates were all similar and there were no major defective areas in the filters andprovided an opportunity for each filter to be washed prior to testing and the azo-dye-functionalization process. A minimum of 15 min of water flushes post-functionalization were also collected and analyzed, helping to characterize the filters. Water flow rates for pre- and post-azo-dye-functionalization during these 15 min periods are included in Supplementary Materials Table S2.

## 3. Results and Discussion

**Figure 6:**
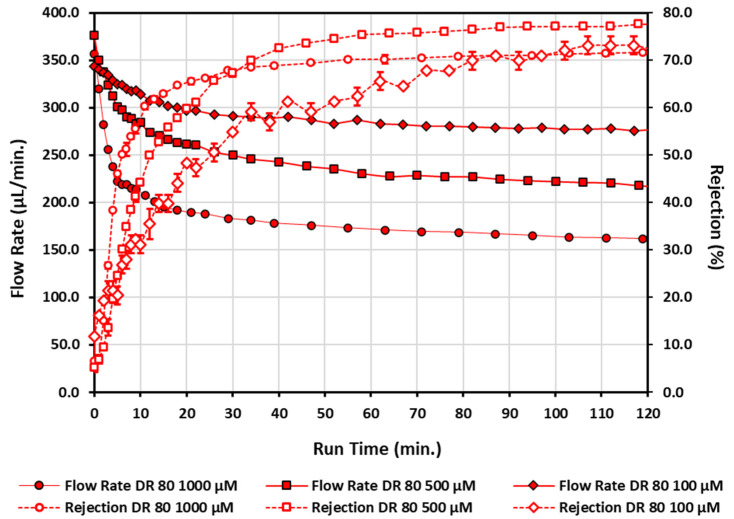
Flow rate and rejection measurements for three different concentrations of direct red 80 as a function of run time. Open data points connected by a dashed line correspond to rejection measurements, while solid data points connected by a solid line corresponds to flow rate measurements. Diamonds—100 µM; squares—500 µM; circles—1000 µM.

Figure 6 illustrates the end results of the azo-dye-functionalization process with direct red 80 for three separate initially unmodified, commercially available polycarbonate filters at three separate concentrations. Error bars for the rejection measurements represent the standard deviation of three rejections measurements made per sample and are typically smaller than the data points for the 500 µM and 1000 µM series, but more noticeable for the 100 µM concentration. Flow rate data is comprised of singular measurements with no error bars. The highest concentration of direct red 80 tested has the greatest reduction in flow rate and the largest functionalization rate. A similar trend is found for the lower concentrations of direct red 80—the lower the concentration, the lower the functionalization rate; however, ultimately, if it is run long enough, then similar rejection values are obtained with different terminal flow rates. Despite the water flow rates prior to functionalization having less than a 0.9% variation from filter to filter from the nominal flow rate, the terminal water flow rates for all three direct red 80 azo-dye-functionalized filters were significantly different after functionalization. At 1000 µM, 500 µM, and 100 µM, the water flow rates post-azo-dye-functionalization had 32.0%, 25.6%, and 13.2% decreases, respectively. Despite the large differences in flow rates, independent of concentration, similar terminal rejection values were achieved within an 8.3% difference. The direct dye molecules certainly flow through the azo-dye-functionalized filter differently than water molecules. For direct red 80, the water flow rates post-azo-dye-functionalization were always higher for the first 15 min of the pure water wash cycle compared with the last 5 measurements of the azo dye being pulled through the polycarbonate filters (data collection time for the 5 combined data points is 25–40 min). The increase in flow rate for water compared with the dye was 13.6% for the 100 µM, 31.7% for the 500 µM, and 55.6% for 1000 µM direct red 80. Despite water flow rates having different terminal values, within the last 15 min of the post-water-wash cycle, the deviation from nominal flow rate values was only between 0.7–2.5% depending upon the concentration tested. This stability of the water flow rate and the ultimate deceases in water flow rates that do not recover to the initial water flow rates prior to functionalization indicate that the azo dye was not washing out, and that the polycarbonate surface, or environment at the surface of the membrane, was permanently changed under such operating conditions.

**Figure 7:**
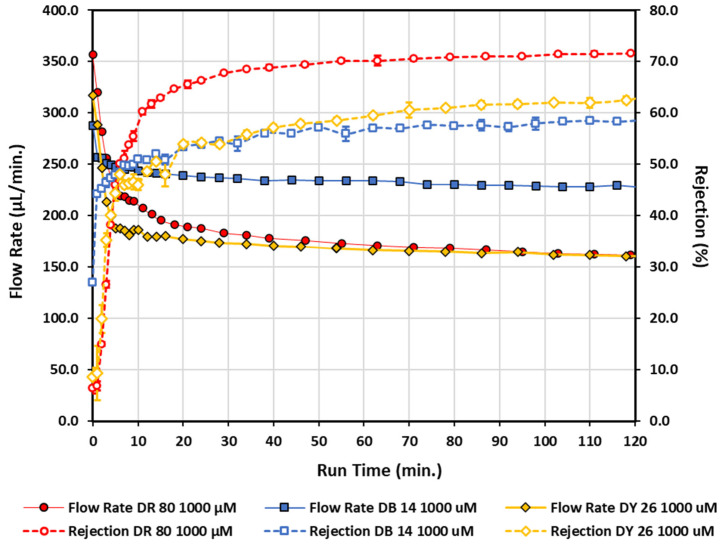
Flow rate and rejection measurements as a function of run time for direct red 80 (same data as in Figure 2), direct blue 14, and direct yellow 26 all at 1000 µM. Open data points connected by a dashed line correspond to rejection measurements, while solid data points connected by a solid line correspond to flow rate measurements. Diamonds—direct yellow 26; squares—direct blue 14; circles—direct red 80.

In Figure 6, it was observed that the concentration of the azo dye changes the functionalization rate. Two other anionic direct azo dye molecules that would have differing charges after dissociation in an aqueous solution were tested for functionalization characteristics. Figure 7 illustrates the end results of the same functionalization process for these two other anionic direct azo dyes at the same concentration of 1000 µM, each on separate initially unmodified polycarbonate filters. Error bars for the rejection measurements represent the standard deviation of three rejections measurements made per sample and are typically smaller than the data points for all dyes tested at this concentration. Flow rate data are comprised of singular measurements with no error bars. The direct red 80 data (1000 µM circles) from Figure 6 are replicated in Figure 7 for comparison. The direct red 80, direct blue 14, and direct yellow 26 all at 1000 µM exhibited water flow rates post-azo-dye-functionalization that had decreases of 32.0%, 26.5%, and 24.3%, respectively, during the 15 min post-wash cycles with water. Similarly, the direct blue 14 and the direct yellow 26 act differently when being pulled through the azo-functionalized filters. The flow rate for water during the first 15 min of the wash cycle compared to the flow rate for dyes for the last 5 measurements of the azo dye being pulled through the polycarbonate filters (data collection time for the 5 combined data points is 30–40 min) increased by 14.2% and 66.1%, respectively. After the 15 min water rinse cycle, the filter used with direct yellow 26 at 1000 µM showed signs of visible discoloration, while the filters used with direct red 80 and direct blue 14 at 1000 µM showed no visible discoloration as a result of the functionalization process, even though they also displayed lower flow rates. In order for the flow rate to stabilize for the direct yellow 26 functionalized filter, it took 80 min of continuous washing to achieve a 15 min flow rate with deviations less than 0.1% from the average flow rate. Only an additional 27 min was needed for direct blue 14 to achieve a 15 min flow rate with deviations of 1.2% from the average flow rate. Even after such excessive washing, the direct yellow 26 filter remained visibly stained yellow. The final small deviations from nominal flow rate values between 0.1–1.2% for both filters during the final 15 min of washing also indicate the modifications to the polycarbonate are permanent and not washing out. Since direct red 80 at 1000 µM had the shortest functionalization time, quickly increasing the rejection to approximately 60% in roughly 10 min, and had the largest rejection response, with no complications with washing out any potentially unbounded dye, it was selected as the primary functionalization fluid for polycarbonate membranes in this study.

Figure 7 also highlights that there was not a systematic increase in rejection with increasing intrinsic charge of the dye molecule as was initially anticipated to be the dominant factor. For example, direct blue 14 and direct yellow 26 differed in the magnitude of intrinsic charge by a factor of two, but both had nearly the same trends and terminal values in rejection within an 8.5% difference. There does not seem to be a systematic correlation based purely on flow rate either, as direct yellow 26, which has an intrinsic charge of negative 2, has a nearly identical terminal flow rate as direct red 80, which has an intrinsic charge of negative 6, but both have notable different rejection values at 11.5% difference despite the very similar terminal flow rates. The trends presented in Figure 7 point to the realization that the intrinsic charge of the dye molecule alone cannot be the sole factor in the observed rejection. Other factors—such as the functionality of the end groups of the negatively charged azo dye molecules used, and how those end groups affect adsorption to the polycarbonate surface—likely play a large role in the rejection and flow rate trends.

**Figure 8:**
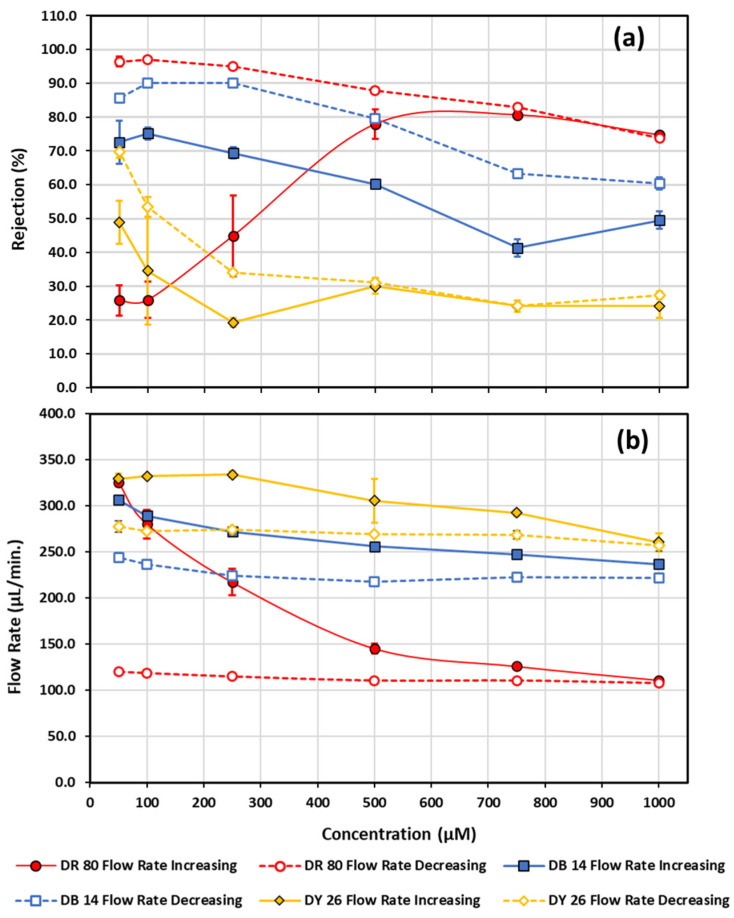
Rejection (**a**) and flow rate (**b**) measurements as a function of increasing and decreasing concentration are shown for direct red 80, direct blue 14, and direct yellow 26. Solid data points connected by a solid line correspond to measurements made going from low to higher concentration, while open data points connected by a dashed line correspond to measurements made while going from high to lower concentration. Diamonds—direct yellow 26; squares—direct blue 14; circles—direct red 80.

Figure 8a illustrates how the azo-dye-functionalization process of polycarbonate at high concentrations can be used to create a filter that is capable of eliminating certain dyes at lower concentrations, equivalent to those concentrations found in textile wastewater streams. Error bars for the rejection and flow rate measurements represent the standard deviation of the last four out of five sample measurements collected per concentration and are typically smaller than the data points for most experiments conducted, with exceptions where functionalization is still occurring. Each dye series functionalization was performed on a fresh unmodified polycarbonate filter that was first washed with water for 15 min prior to functionalization. Figure 6 and Figure 7 demonstrate that using 1000 µM direct red 80 increases the rejection to roughly 60% in 10 min and only increases by another ~10% over the next 110 min; therefore, for each concentration, 5 consecutive 3-minute measurements of flow rate and rejection were made, analyzing the last 4 of those measurements to create Figure 8. The concentration was first ramped up then ramped back down in concentration, with three consecutive 5 min water runs through the filter between each concentration to flush out unbound molecules. Any hysteresis as the concentration is ramped up and back down can easily be attributed to some degree of functionalization of the original polycarbonate membrane. The minimum dye concentration tested was chosen to be 50 µM, which is in close agreement with typical effluent concentrations encountered in textile dyeing operations [101]. Figure 8 further exemplifies that an unmodified polycarbonate is highly susceptible to functionalization with not only direct red 80, but also with direct blue 14 and direct yellow 26, with each dye having different rejection responses. Direct yellow 26 seemed to quickly plateau in terms of rejection, with some minimal hysteresis value towards the lower concentrations, while the direct blue 14 rejection actually decreased in rejection with increasing concentration, yet provided a more constant offset in rejection as the concentration was lowered. Direct red 80 behaved nothing like the other two dyes. It first drastically increased in rejection and then leveled off and started to decrease in rejection as the concentration was increased; however, when direct 80 was decreased in concentration, the rejection continued to climb, approaching near 100% (96.4 ± 1.4%) rejection as the flow rate remained steady at (114 ± 5) µL/min from 1000 µM down to 50 µM. Figure 8b shows the flow rates as a function of concentration and highlights that the near 100% rejection of the 50 µM direct red 80 occurred at nearly the same flow rate as the 1000 µM sample, providing a very stable flow rate. In fact, all the flow rate data in Figure 8b, shown by the dashed lines, demonstrates that, after the max functionalization was reached at the max concentration tested (1000 µM), the flow rate of the filters for all 3 dyes became very stable as lower concentrations were reached. Having a filter that has a stable flow rate, while successfully filtering at your desired concentration, is highly desirable. The success demonstrated in Figure 8 with 50 µM warrants further investigation. Two azo-dye-functionalized filters in succession should have no issues in easily turning the 96.4% rejection of 50 µM direct red 80 azo molecular dye into 100% rejection, while keeping the same flow rate.

**Figure 9:**
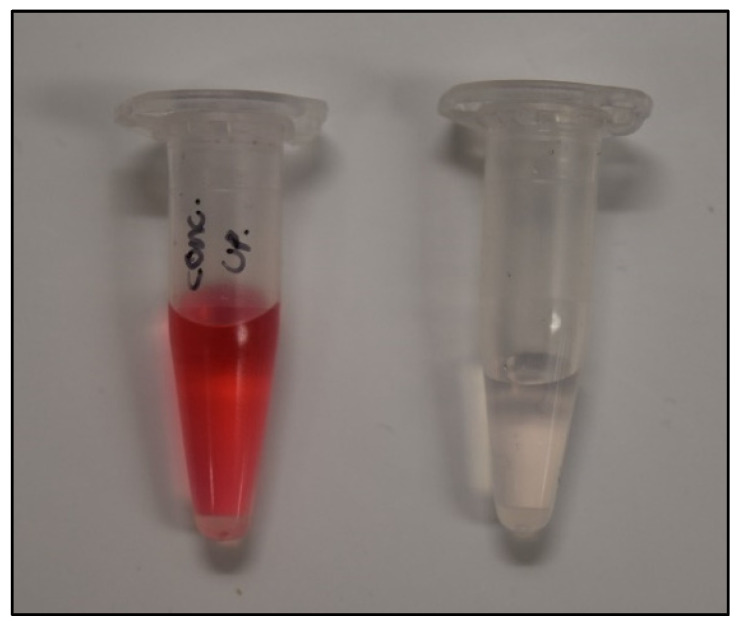
A measure of 50 µM direct red 80 feed solution (**left**) versus 50 µM direct red 80 permeate solution (**right**) that has been run through an azo-functionalized polycarbonate filter, resulting in (96.4 ± 1.4%) rejection.

Frequently numbers such as 50 µM and 1000 µM for concentrations of various solutions are found in textbooks, publications, and transparent solutions and sometimes we need a physical reminder of their significance. In the context of textile dyes, 50 µM is highly colored and is at the approximate concentration of textile effluents. The impressive and dramatic near 100% rejection of 50 µM direct red 80 azo molecular dye is shown in Figure 9. The vial on the right contains the permeate of the final decreasing concentration run at 50 µM, shown in Figure 8a, near 100% rejection, and the vial on the left is the 50 µM feed solution fed into the already azo-dye-functionalized filter.

**Figure 10:**
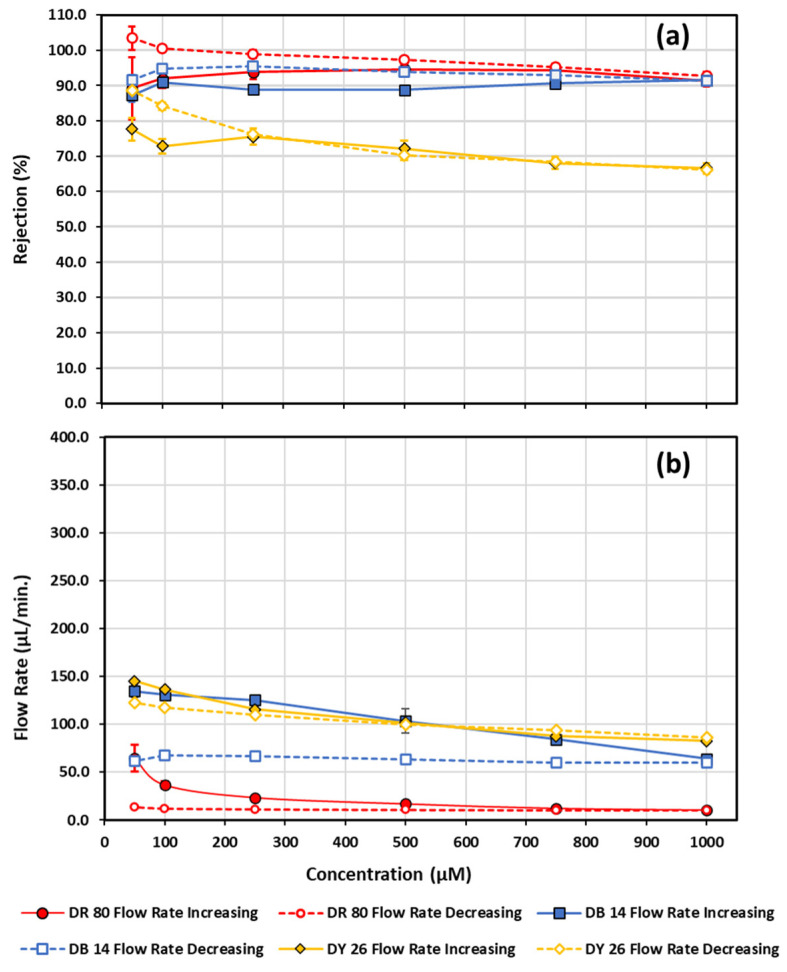
Rejection (**a**) and flow rate (**b**) measurements as a function of increasing and decreasing concentration for direct red 80, direct blue 14, and direct yellow 26. Closed data points connected by a solid line correspond to measurements made going from low to higher concentration, while open data points connected by a dashed line correspond to measurements made while going from high to lower concentration. Diamonds—direct yellow 26; squares—direct blue 14; circles—direct red 80.

How do these azo dye functionalized polycarbonate filters compare to recent state-of-the-art filtration technologies? To present a comparison, recently developed ultrathin self-assembled nanoparticle (USANP) membranes, prepared on the exact same polycarbonate support, using similar, but not identical preparation methods to the literature [79,80], were subject to the same exact same azo-dye-functionalization as unmodified polycarbonate filters, as shown in Figure 8. Figure 10 illustrates the results of the azo-dye-functionalization process on the polycarbonate filters which already have 6–8 monolayers of porous USANPs deposited. Error bars for the rejection and flow rate measurements represent the standard deviation of the last four out of five sample measurements collected per concentration and are typically smaller than the data points for most experiments conducted, the exceptions being where functionalization is still occurring. Figure 10a shows that these nanoparticle-functionalized filters have an impressive level of rejection that spans the concentration range tested; however, Figure 10b shows that this increased rejection over a wider concentration range comes at the cost of a much lower flow rate compared with the azo-dye-functionalized filters by over an order of magnitude. Figure 10b shows that, as the concentration increases to 1000 µM, the flow rate drops from an already low 64.7 µL/min to 10.0 µL/min, and never recovers when the concentration is decreased. It is interesting to note that the gold nanoparticle monolayers seem to prevent the polycarbonate filter from achieving a large amount of functionalization, as the hysteresis values in rejection between increasing and decreasing concentration are lower compared with the azo-dye-functionalization of the bare polycarbonate filters in Figure 8a; however, some permanent azo-dye-functionalization is still possible for the already USANP-functionalized filters. In summary, Figure 8, Figure 9 and Figure 10 show that the azo-functionalized polycarbonate filters can reject 50 µM molecular dyes with a high rejection rate and have a higher stable flow rate compared with polycarbonate filters using porous USANP monolayers, which have significantly lower flow rates but higher rejection rates over a wider range of concentrations.

How do the filters, functionalized with the azo dye direct red 80, affect other dyes at wastewater concentrations? To answer this question, 50 µM concentrations of direct yellow 26, direct blue 14, and direct red 80 were first run through an unmodified polycarbonate filter in that order, followed by a 35 min in situ azo-dye-functionalization with 1000 µM direct red 80. Then, 50 µM concentrations of direct red 80, direct blue 14, and direct yellow 26 (in that specific order) were run again all on the same filter. Each dye fluid was analyzed over 15 min. The 15 min water wash runs were also carried out between each dye and pre- and post-functionalization. In the end, the average flow rate for the dyes had a decrease of 32.1%. The rejection for direct yellow 26, direct blue 14, and direct red 80 had increases of 53.2%, 32.1%, and 26.3%, respectively. Table 1 highlights the effects of the azo-dye-functionalization on an initially unmodified polycarbonate filter. Uncertainties for the rejection and flow rate measurements represent the standard deviation of the last four out of five sample measurements collected.

Table 2 illustrates the effects of running 50 µM concentrations of direct yellow 26, direct blue 14, and direct red 80 on an already functionalized USANP filter that was then further azo-dye-functionalized with 1000 µM direct red 80 for 35 min, followed by running the same 50 µM concentrations of direct red 80, direct blue 14, and direct yellow 26 (in that specific order). Uncertainties for the rejection and flow rate measurements represent the standard deviation of the last four out of five sample measurements collected. Each dye fluid was analyzed over 15 min. The 15 min water wash runs were carried out between each dye. In the end, the flow rate for the dyes decreased by 43.4%, 37.0%, and 28.1% for direct yellow 26, direct blue 14, and direct red 80, respectively. The rejection for direct blue 14 and direct red 80 increased by only 1.0% and 2.1%, respectively. The effects of the azo-dye-functionalization on the USANP-modified filters are clearly seen from the first direct yellow 26, run through the filter. When 50 µM direct blue 14 and direct red 80 were run before and after the direct red 80 functionalization run, the direct yellow 26 had an increase in rejection of 20.5% when retested.

Table 3 demonstrates the effects that the 1000 µM direct red 80 azo-dye-functionalization has on the rejection of both the positive sodium ions and negatively charged nitrate ions in a solution that passes through an initially unmodified polycarbonate filter. Uncertainties for the rejection and flow rate measurements represent the standard deviation of the four measurements collected. First, sodium nitrate salt solutions were made at 250, 500, and 750 µM. They were then filtered through an unmodified polycarbonate filter which showed virtually no rejection when averaged as a group. Next, the azo-dye-functionalization was completed with the 1000 µM direct red 80 azo dye for 40 min in situ, as in the other experiments. Then, the ion solutions were rerun. Post-azo-dye-functionalization, sodium, and nitrate ions had, on average, 17.9%, 26.0%, and 31.1% rejection for the 750, 500, and 250 µM solutions, respectively, at an average flow rate of 177 ± 5 µL/min post-functionalization. The 15 min water wash runs were carried out between each sodium nitrate concentration test and pre- and post-dye functionalization. The observed average decrease in flow rate for the electrolyte solution pre- and post-dye functionalization was 48.0%.

Table 4 emphasizes the effects that the 1000 µM direct red 80 azo-dye-functionalization had on the rejection of both the positive sodium ions and negatively charged nitrate ions in a solution that passes through a filter that has already been prefunctionalized with USANP monolayers. Uncertainties for the rejection and flow rate measurements represent the standard deviation of four measurements collected. First, the sodium nitrate salt solutions were filtered through a USANP-functionalized polycarbonate filter, which showed an average range in rejection of 5–18%. Next, the azo-dye-functionalization was completed with the 1000 µM direct red 80 azo dye for 40 min in situ as in the other experiments. Then, the ion solutions were rerun. Post-azo-dye-functionalization, sodium and nitrate ions showed an increase in the rejection in the range of 30–50%. The 15 min water wash runs were carried out between each sodium nitrate concentration test and pre- and post-dye functionalization. The observed average decrease in flow rate is 57.2% for the electrolyte solution after dye-functionalization.

These dye molecules are only a few nanometers in their largest dimension, although they are much larger than the sodium and nitrate ions tested, and are much smaller than the 100 nm-diameter pores in the polycarbonate filter. So, why do they not simply go straight through the pores in the polycarbonate filter? It is evident from the direct yellow 26 functionalization tests (Figure 7) the dye is capable of staining the polycarbonate filter and that there is some degree of permanent adsorption or attachment of the dyes to the polycarbonate filter. Such modifications are likely happening to some extent with the direct red 80 and direct blue 14, even if the observer cannot see it as a strong visual discoloration. The direct yellow 26 does not have sulfonate groups as end groups, but rather has carboxylate groups. This change in the end group might have been responsible for the stronger adsorption to the surface and may also have affected any systematic trends with the sulfonated dyes in Figure 7. It would be interesting to see how a sulfonated azo dye with an intrinsic-2 charge would behave. This adsorption or attachment of the dye molecule likely has two effects that increase the rejection of charged contaminates. Firstly, the adsorption or attachment of a dye molecule to the polycarbonate potentially adds an additional six units of charge to the membrane via the six sulfonate groups, with each direct red 80 molecule that attaches. Since the membrane is then charged, a Donnan potential at the surface of the membrane would exist, and, when combined with an electroneutrality requirement, would aid in rejecting both the co-ions and counter ions in any electrolyte solution filtered [102]. Table 3 and Table 4 certainly support this hypothesis, because both the positive sodium and negative nitrate ions have very similar rejections post-azo-dye-functionalization. Based on the unmodified pore size of the commercial polycarbonate filters being 100 nm in diameter, they would be classified as borderline ultrafiltration/microfiltration, with any surface modification by the azo dyes, only decreasing the effective pore size.

The ending rejection for 50 µM direct red 80 in Table 1 is not as high as in Figure 8, most likely because the direct yellow 26 and direct blue 14, which were run first, were not the dyes to maximally functionalize the polycarbonate filter; meaning they likely attached and occupied binding sites on the polycarbonate that could have been occupied by direct red 80 molecules, which may have deposited more charge on the polycarbonate, effecting rejection through the surface charge. Alternatively, and more simply, more direct red 80 dye was run through the filter at various concentrations, as shown in Figure 8, which could be responsible for the increased rejection. Secondly, the adsorption or attachment of the dye molecule to the polycarbonate surface could potentially reduce the radius of the pore diameter if the molecules were lining the inner walls; alternatively, the dye molecules may even be forming a gel or cake layer at the filter surface, where an effective pore size may become smaller, leading to increased rejection from steric hindrance factors [102,103]. With water and dye flow rates reduced by over 60% in some cases as rejection increases—as indicated in Table 1, Table 2, Table 3 and Table 4, Supplementary Table S1, and Figure 6, Figure 7 and Figure 8—fouling mechanisms may play a crucial role in the success of these filters. Such a fouling event for most filters would be seen as detrimental to their performance; however, here, it may be a requirement for the performance of such azo-dye-functionalized filters. The type of fouling likely depends on the concentration of the functionalization fluid, because terminal flow rates were different, as shown in Figure 6, with different concentrations of the same fluid for functionalization, but end with similar terminal rejections over the tested time period.

The exact forces that potentially bind the azo dye molecules to the polycarbonate needs more investigation. Additionally, quantification in several areas, including the amount of dye bound to the polycarbonate, the specific type of fouling, and the lifetime of any developed gel/cake layers, is needed. Understanding how the potentially adsorbed molecules and fouling layers influence the surface charge may help to optimize such azo-dye-functionalized filters. There is much that can be expanded on in this work. For example, we ask the following questions: Can the azo dye’s adhesion to the polycarbonate be increased by using mordant additives, such as salts, by increasing the temperature, or by varying the pH during or prior to functionalization, as is carried out in the industrial processes for fabrics? In fact, would other dyes other than azo dyes provide better rejection results at higher flow rates, because they might have better adhesion to the polycarbonate? Future testing needs to be carried out under high salt concentrations to mimic more realistic dying scenarios in the industry. The polycarbonate filters used here are commercially available, but are limited in size and porosity; however, to scale up the process and increase the flow rate, larger and more porous polycarbonate membranes should be possible. Such commercial filters are typically cut from larger rolls, which are exposed to charged particles in nuclear or heavy-ion reactors; if they are exposed for longer times during the track-etching process, then we would observe larger porosity and increased flow rate [104,105].

## 4. Conclusions

In summary, it has been shown that porous polycarbonate membranes, when functionalized with azo textile dyes themselves, can provide a new method for the successful removal and decolorization of textile dyes from water. The success of such filters is not limited to azo dyes: after the azo-dye-functionalization process, rejection increases for charged contaminates—such as the sodium and nitrate ions found in other industrial waste streams, e.g., the agricultural industry are observed. Data shows that, once functionalized, these filters are less susceptible to fouling over time and leave the azo bond intact pre- and post-filtering, overcoming some of the current challenges for membrane filtration of textile dyes. The rejection values at the concentrations of interest compare well with the state-of-the-art ultrathin self-assembled nanoparticle monolayers, with an added benefit—the flow rate is an order of magnitude higher. The simplistic nature of this azo-dye-functionalization technique provides an elegant solution to the problem of the pollution of freshwater by textile dyes, demonstrating that the textile dyes, and thus the contaminants themselves, are part of the solution to the problem.

## Figures and Tables

**Table 1:** Rejection and flow rate values for 50 µM concentrations of direct yellow 26, direct blue 14, and direct red 80 pre- and post-azo-dye-functionalization on an unmodified polycarbonate filter with direct red 80 at 1000 µM for 35 min.

	Conc. (µM)	Flow Rate (µL/min)	Rejection (%)
DY 26	50	291.5 ± 7.1	53.3 ± 3.7
DB 14	50	292.7 ± 0.6	71.2 ± 1.5
DR 80	50	280.9 ± 5.0	69.5 ± 10.5
1000 µM DR 80 Functionalization			
DR 80	50	192.4 ± 1.0	87.8 ± 5.8
DB 14	50	198.9 ± 1.4	94.0 ± 1.7
DY26	50	196.3 ± 1.8	81.6 ± 2.1
		% Dec. In Flow Rate (%)	% Inc. in Rejection (%)
DR 80	50	31.5	26.3
DB 14	50	32.0	32.1
DY26	50	32.7	53.2

**Table 2:** Rejection and flow rate values for 50 µM concentrations of direct yellow 26, direct blue 14, and direct red 80 pre- and post-azo-dye-functionalization, using direct red 80 at 1000 µM for 35 min on a polycarbonate filter already prefunctionalized with USANP monolayers.

	Conc. (µM)	Flow Rate (µL/min)	Rejection (%)
DY 26	50	169.5 ± 3.3	77.3 ± 1.3
DB 14	50	157.1 ± 3.7	93.5 ± 1.2
DR 80	50	125.6 ± 6.3	96.9 ± 2.4
1000 µM DR 80 Functionalization			
DR 80	50	90.2 ± 3.3	98.9 ± 0.9
DB 14	50	98.9 ± 2.6	94.5 ± 0.5
DY26	50	96.0 ± 2.2	93.1 ± 1.4
		% Dec. in Flow Rate (%)	% Inc. in Rejection (%)
DR 80	50	28.1	2.1
DB 14	50	37.0	1.0
DY26	50	43.4	20.5

**Table 3:** Rejection and flow rate values for 250, 500, and 750 µM concentrations of sodium nitrate solution pre- and post-azo-dye-functionalization, on an unmodified polycarbonate filter, with direct red 80 at 1000 µM for 40 min.

Conc. (µM)	Na-Rejection (%)	NO_3_-Rejection (%)
250	0.0 ± 0.0	−6.2 ± 4.2
500	2.3 ± 4.5	3.8 ± 3.1
750	−3.1 ± 3.6	−0.8 ± 5.3
1000 µM DR 80 Functionalization		
750	17.6 ± 4.8	18.1 ± 7.0
500	25.0 ± 4.5	27.0 ± 3.8
250	33.3 ± 0.0	28.8 ± 28.2
	NaNO_3_-Flow Rate% Dec.	
250	45.6	
500	47.2	
750	51.3	

**Table 4:** Rejection and flow rate values for 250, 500, and 750 µM concentrations of sodium nitrate solution pre- and post-azo-dye-functionalization, using direct red 80 at 1000 µM for 40 min on a polycarbonate filter, already prefunctionalized with USANP monolayers.

Conc. (µM)	Na-Rejection (%)	NO_3_-Rejection (%)
250	17.9 ± 7.1	16.7 ± 4.5
500	11.1 ± 4.8	10.7 ± 6.1
750	4.7 ± 3.1	8.8 ± 4.6
1000 µM DR 80 Functionalization		
750	31.3 ± 0.0	35.9 ± 2.6
500	30.0 ± 8.2	33.3 ± 6.8
250	50.0 ± 0.0	37.5 ± 4.8
	NaNO_3_-Flow Rate% Dec.	
250	53.1	
500	58.1	
750	60.3	

## Data Availability

All data presented in this study are openly available in the Marshall Digital Scholar repository. Attention is drawn to only a few outliers that were collected, but excluded from analysis, as the rejection or flow rate values were seemingly drastically out of place with shown trends. These outliers have likely resulted from errors in the collection protocols (recirculation values closed on filtration chamber, peristaltic pump off, etc.), and transcription errors or evident complications were noted, and/or otherwise have no explanation, but are included in the data file for completeness.

## Supplementary Materials

The following supporting information can be downloaded at: https://www.mdpi.com/article/10.3390/mi13040577/s1, Video S1: Large-scale concentration gradient removal—Side View, Video S2: Large-scale concentration gradient removal—Top View. Figure S1: Absorbance as a function of concentration for azo dyes, Figure S2: Sodium and nitrate ion concentration as a function of sodium nitrate concentration, Table S1: Feed concentrations for all 1000 µM functionalization trials; Table S2: Water flow rate characterization.
