# Supplementary material for: Azo-Dye-Functionalized Polycarbonate Membranes for Textile Dye and Nitrate Ion Removal
Source: Micromachines (Basel). 2022 Apr 7;13(4):577. doi: 10.3390/mi13040577 (PMC9030370; doi:10.3390/mi13040577)
Supplement: Supplementary file 1 [file micromachines-13-00577-s001.zip › supplementary.pdf]

micromachines

MDPI

Supplementary Material

Azo-Dye-Functionalized Polycarbonate Membranes for Textile Dye and Nitrate Ion Removal

Carrie Cockerham [1], Ashton Caruthers [2,3], Jeremy McCloud [4,5], Laura M. Fortner [4,6], Sungmin Youn [1] and Sean P. McBride [5,*]

1 Department of Civil Engineering, Marshall University, Huntington, WV 25755, USA; cockerham2@marshall.edu (C.C.); youns@marshall.edu (S.Y.)
2 John T. Hoggard High School, 4305 Shipyard Boulevard, Wilmington, NC 28403, USA; ashton.caruthers@nhcs.net
3 Department of Curriculum Instruction & Foundations, Marshall University, Huntington, WV 25755, USA
4 Department of Mechanical Engineering, Marshall University, Huntington, WV 25755, USA; mccloud54@marshall.edu (J.M.); fortner40@marshall.edu (L.M.F.)
5 Department of Physics, Marshall University, Huntington, WV 25755, USA
6 Spring Valley High School, 1 Timberwolf Drive, Huntington, WV 25704, USA
* Correspondence: mcbrides@marshall.edu; Tel.: +1-304-696-2758

Video S1. Large scale concentration gradient removal - Side View ().

Video S2. Large scale concentration gradient removal - Top View (link).

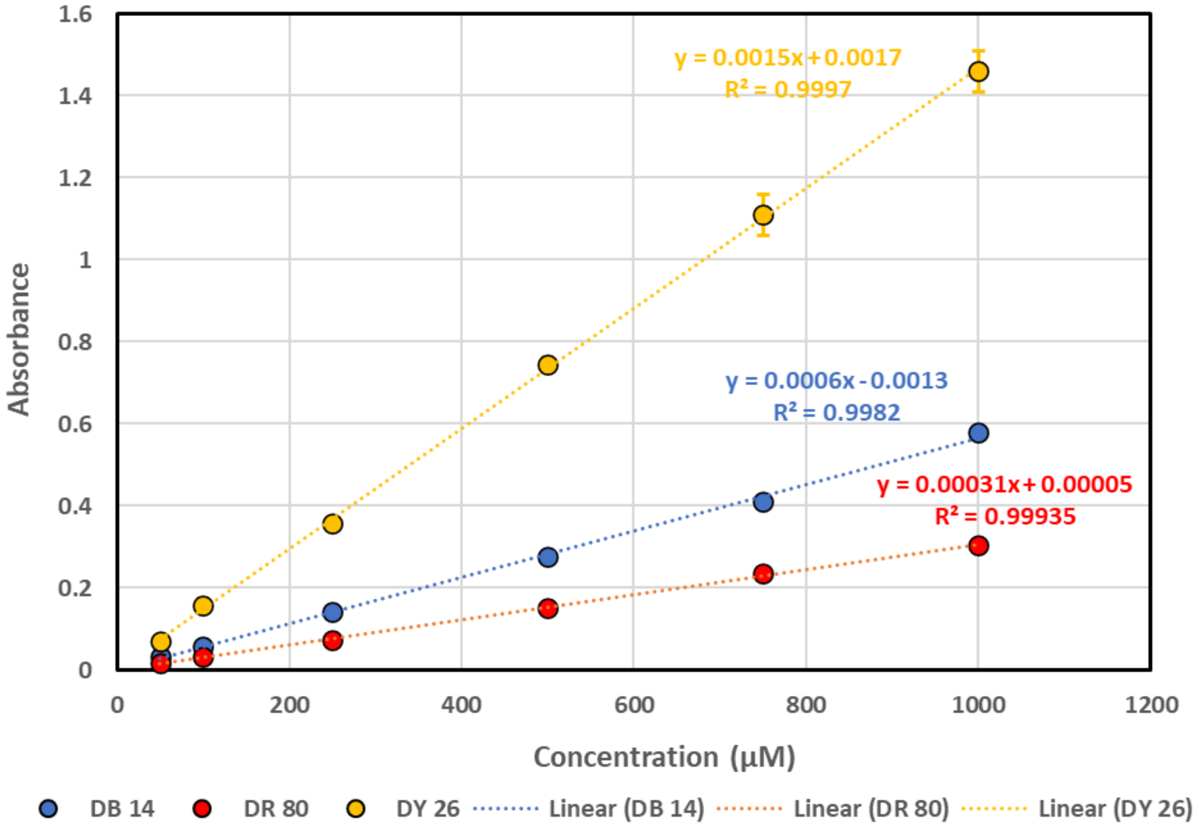

Figure S1. Ultraviolet visible light spectroscopy absorbance of azo dye solutions, tested as a function of concentration.

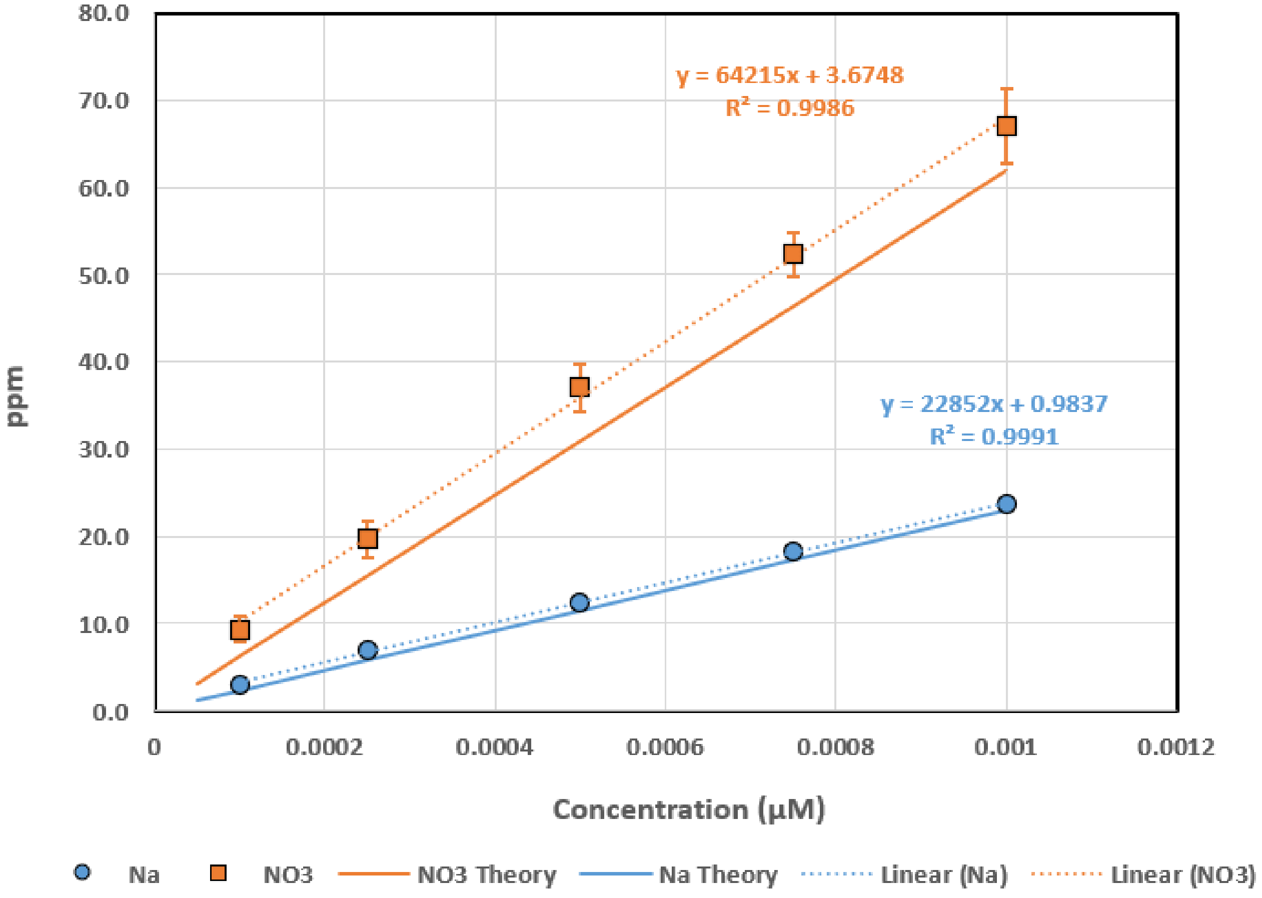

Figure S2. Sodium and nitrate ion concentration as a function of sodium nitrate solution concentration.

Table S1. Concentration of direct red 80 for all 1000 µM feed solutions over the functionalization experiments.

**Absorbance of feed for all 1000 µM experiments (no systematic change in feed concentration detectable)**									
**1**	**0.300**	**0.306**	**0.301**	**0.308**	**0.304**	**0.305**	**0.302**	**0.310**	0.306
2	0.304	0.307	0.303	0.314	0.307	0.310	0.309	0.320	0.313
3	0.308	0.310	0.305	0.315	0.308	0.314	0.318	0.327	0.310
Ave	0.304	0.308	0.303	0.312	0.306	0.310	0.310	0.319	0.310
Δ	0.004	0.002	0.002	0.004	0.002	0.005	0.008	0.009	0.004
%	1.3	0.7	0.7	1.2	0.7	1.5	2.6	2.7	1.1

Table S2. Data for 15-minute water flushes pre- and post-functionalization.

H_2O Pre-Flow Rate (µl/min.)	Δ (µl/min.)	%	Functionalization Fluid	Concentration (µM)	H_2O Post Flow Rate (µl/min.)	Δ (µl/min.)	%	% Decrease
Initially Unmodified Polycarbonate Filter Water Flow Rates Pre- and Post-Functionalization (first 15 minutes)								
375.3	2.7	0.7	DR 80	1000	255.1	5.5	2.1	32.0
389.1	0.2	0.1	DR 80	500	289.5	7.1	2.5	25.6
362.8	2.4	0.7	DR 80	100	314.8	2.0	0.7	13.2
354.9	1.1	0.3	DB 14	1000	260.9	12.0	4.6	26.5
355.4	1.1	0.3	DY 26	1000	268.9	6.1	2.3	24.3
372.3	3.3	0.9	DR 80	50-1000	142.1	1.4	1.0	61.8
372.3	3.3	0.9	DB 14	50-1000	296.4	1.8	0.6	20.4
380.2	1.1	0.3	DY 26	50-1000	290.7	5.3	1.8	23.5
354.4	1.0	0.3	DR 80, DB 14, DY 26	50-1000	245.7	1.1	0.4	30.7
356.5	1.8	0.5	DR 80	1000	203.9	1.7	0.8	42.8
Gold-Nanoparticle-Modified Polycarbonate Filters Water Flow Rates Pre- and Post-Functionalization (first 15 minutes)								
126.5	3.2	2.5	DR 80	50–1000	17.1	0.8	4.9	86.5
177.3	6.7	3.8	DB 14	50–1000	70.1	1.5	2.1	60.5
174.2	3.2	1.8	DY 26	50–1000	146.0	2.3	1.6	16.2
190.6	3.0	1.6	DR 80, DB 14, DY 26	50–1000	121.3	1.5	1.2	36.4
210.1	4.3	2.0	DR80	1000	107.1	0.9	0.8	49.0
